# Citrullination of C1-inhibitor as a mechanism of impaired complement regulation in rheumatoid arthritis

**DOI:** 10.3389/fimmu.2023.1203506

**Published:** 2023-06-22

**Authors:** Myriam Martin, Sara C. Nilsson, David Eikrem, Karin Fromell, Carsten Scavenius, Leonie M. Vogt, Ewa Bielecka, Jan Potempa, Jan J. Enghild, Bo Nilsson, Kristina N. Ekdahl, Meliha C. Kapetanovic, Anna M. Blom

**Affiliations:** ^1^ Department of Translational Medicine, Division of Medical Protein Chemistry, Lund University, Malmö, Sweden; ^2^ Department of Immunology, Genetics and Pathology, Rudbeck Laboratory, Uppsala University, Uppsala, Sweden; ^3^ Department of Molecular Biology and Genetics, Aarhus University, Aarhus, Denmark; ^4^ Malopolska Centre of Biotechnology, Jagiellonian University, Krakow, Poland; ^5^ Department of Microbiology, Faculty of Biochemistry, Biophysics and Biotechnology, Jagiellonian University, Krakow, Poland; ^6^ Department of Oral Immunology and Infectious Diseases, School of Dentistry, University of Louisville, Louisville, KY, United States; ^7^ School of Natural Sciences, Linnæus University, Kalmar, Sweden; ^8^ Department of Clinical Sciences Lund, Rheumatology, Lund University, Skåne University Hospital, Lund, Sweden

**Keywords:** citrullination, C1-inhibitor, complement system, PAD, rheumatoid arthritis, synovial fluid, ACPA

## Abstract

**Background:**

Dysregulated complement activation, increased protein citrullination, and production of autoantibodies against citrullinated proteins are hallmarks of rheumatoid arthritis (RA). Citrullination is induced by immune cell-derived peptidyl-Arg deiminases (PADs), which are overactivated in the inflamed synovium. We characterized the effect of PAD2- and PAD4-induced citrullination on the ability of the plasma-derived serpin C1-inhibitor (C1-INH) to inhibit complement and contact system activation.

**Methods:**

Citrullination of the C1-INH was confirmed by ELISA and Western blotting using a biotinylated phenylglyoxal probe. C1-INH-mediated inhibition of complement activation was analyzed by C1-esterase activity assay. Downstream inhibition of complement was studied by C4b deposition on heat-aggregated IgGs by ELISA, using pooled normal human serum as a complement source. Inhibition of the contact system was investigated by chromogenic activity assays for factor XIIa, plasma kallikrein, and factor XIa. In addition, autoantibody reactivity to native and citrullinated C1-INH was measured by ELISA in 101 RA patient samples.

**Results:**

C1-INH was efficiently citrullinated by PAD2 and PAD4. Citrullinated C1-INH was not able to bind the serine protease C1s and inhibit its activity. Citrullination of the C1-INH abrogated its ability to dissociate the C1-complex and thus inhibit complement activation. Consequently, citrullinated C1-INH had a decreased capacity to inhibit C4b deposition *via* the classical and lectin pathways. The inhibitory effect of C1-INH on the contact system components factor XIIa, plasma kallikrein, and factor XIa was also strongly reduced by citrullination. In RA patient samples, autoantibody binding to PAD2- and PAD4-citrullinated C1-INH was detected. Significantly more binding was observed in anti-citrullinated protein antibody (ACPA)-positive than in ACPA-negative samples.

**Conclusion:**

Citrullination of the C1-INH by recombinant human PAD2 and PAD4 enzymes impaired its ability to inhibit the complement and contact systems *in vitro*. Citrullination seems to render C1-INH more immunogenic, and citrullinated C1-INH might thus be an additional target of the autoantibody response observed in RA patients.

## 1 Introduction

The complement system plays a detrimental role in rheumatoid arthritis (RA) (1). One of the underlying mechanisms is that abundant immune complexes of immunoglobulins and their cognate autoantigens activate the classical complement pathway in the synovium (2, 3). Post-translationally modified proteins and peptides form a specific category of disease-inducing autoantigens in RA. Anti-citrullinated protein antibodies (ACPAs) are present in approximately 75% of RA patients and are among the best markers for RA diagnosis (4). ACPA can be IgG, IgA, or IgM in nature and can be detected in the synovial fluid as well as in serum, and its level correlates with increased disease severity (5, 6). Interestingly, ACPA is present in patient serum already 5 years before the onset of symptoms and can thus function as a predictor of RA (7). Currently, the presence of ACPAs in RA patients is measured by cyclic citrullinated peptide (CCP) assays that use a mixture of cyclized citrullinated peptides as synthetic mimics of true antigens such as fibrinogen, α-enolase, vimentin, filaggrin, and histones (8).

Citrullination by peptidyl-Arg deiminase (PAD) enzymes causes an Arg to be converted into citrulline by replacing the primary ketamine (=NH) with a ketone (=O) (9–11). This causes a net change in molecular charge, from positive at a physiological pH to neutral, which increases its hydrophobicity and can thereby affect protein folding, interactions, and function. Increased expression and activity of PAD2 and PAD4 have been reported in RA patients (12, 13). Human neutrophils, in particular, are known to overexpress PAD enzymes (12), which depend on a reducing environment (14) and relatively high calcium concentrations (15) for their activity.

In addition to typical local synovial proteins, many proteins and inhibitors of the complement system are susceptible to post-translational modifications (7, 16). C1-INH is an acute-phase protein that is mainly produced by hepatocytes, but other cell types, including peripheral blood monocytes, microglia, fibroblasts, endothelial cells, the placenta, and megakaryocytes, can also synthesize and secrete the protein (17). As an important serine protease inhibitor (*SERPING1* gene) (18), its main function in the complement system is to prevent spontaneous activation. C1-INH is present in the blood at an average concentration of 0.25 g/L and can rise ~2-fold during inflammation (19, 20). It is the only natural inhibitor of the two serine proteases C1r and C1s, which together with C1q compose the first component of the classical complement pathway (21). Additionally, C1-INH is a major inhibitor of mannose-associated serine protease (MASP)-1 and MASP-2 of the mannose-binding lectin (MBL) complex, the initial component of the lectin pathway (22). As such, C1-INH prevents the proteolytic cleavage of C4 and C2 by C1 and MBL–MASP complexes (21, 22). In both pathways, binding is thought to be irreversible. Notably, C1-INH is also a dominant inhibitor of the contact system influencing the kallikrein–kinin and fibrinolytic pathways as well as coagulation (23). All components of the kallikrein–kinin system have been found in the synovial fluid of RA patients, and since kinins are important primary mediators of inflammation, it is believed that they play a role in the pathogenesis of RA (24).

Since there is a high level of complement activation in the rheumatoid joint, the presence of activators such as immune complexes, cell debris, and damaged proteins might coincide with a local functional defect of C1-INH. Like many other serpins, C1-INH has a P1-Arg in its reactive center loop, which confers a typical susceptibility to citrullination (7). Because of its position, this Arg is vital to the proper functioning of the protein. Recently, more than 100 citrullinated proteins of which 17% were serine protease inhibitors were identified in the synovial fluid of RA patients. Among the citrullinated serpins was C1-INH, and it was shown that citrullination abolished its ability to inhibit kallikrein (7).

To further explore the consequence of citrullination on the inhibitory effect of C1-INH in the contact system and more importantly in the complement system, we studied the mechanism of how citrullination might impair its function.

## 2 Materials and methods

### 2.1 Healthy control subjects, RA patients, and ethics

Healthy controls were recruited at the Department of Translational Medicine, Lund University, Malmö, and signed informed consent was obtained. Blood samples were collected from RA patients at their regular follow-up visits at the Department of Rheumatology, Skåne University Hospital in Lund. Data on inflammatory markers such as C-reactive protein (CRP) and erythrocyte sedimentation rate (ESR), number of swollen and tender joints using 28 joint count index, physicians’ and patients’ assessment of disease activity, patients’ assessment of pain, and their global health are routinely collected in the Swedish Rheumatology Quality Register (SRQ), which is a part of the rheumatology routine care in Sweden (25). Based on these data, the SRQ automatically calculates the activity in the disease using the activity index (DAS28) (26) and patients’ physical function using the health assessment questionnaire (HAQ) (27). The presence of IgM-rheumatoid factor (RF) and anti-cyclic citrullinated peptide autoantibodies (anti-CCPs) was determined by EliA-method (EliA RF IgM Well and EliA CCP Well, Phadia, Thermo Fisher Scientific, Waltham, MA, USA). All samples were collected in accordance with the Declaration of Helsinki, and the study was approved by the Ethics Committee, Lund University, Sweden (Dnr 2013/846, Dnr 2017/582, Dnr 2021-03923). The demographics of all participants are shown in **Table 1**.

**Table 1:** Demographics and clinical characteristics of RA patients.

Characteristics	Total n = 101
Age at sampling, median (range)	61 (42–78)
Sex (n)	Female (n = 82) Male (n = 19)
Disease duration, median (range)	14 (0.5–51)
Smoking (n)	Never (n = 28) Smoked (n = 52) Smoking (n = 11) ND (n = 10)
ACPA status (n)	Positive (n = 77) Negative (n = 24)
RF status (n)	Positive (n = 73, ACPA+ n = 65, ACPA− n = 8) Negative (n = 24, ACPA+ n = 9, ACPA− n = 15) ND (n = 4, ACPA+ n = 3, ACPA− n = 1)
CRP at sampling, median (range)	7.1 (0–98)
ESR at sampling, median (range)	22 (2–119)
DAS28, median (range)	4.53 (1.19–8.23)
HAQ, median (range)	1.13 (0–9)
Cortisone treatment (n)	Yes (n = 58) No (n = 43)

RA, rheumatoid arthritis; ACPA, anti-citrullinated protein antibody; RF, rheumatoid factor; CRP, C-reactive protein; ESR, erythrocyte sedimentation rate; HAQ, health assessment questionnaire; ND, not determined; ACPA+, ACPA-positive; ACPA−, ACPA-negative.

### 2.2 Proteins

Pharmaceutical-grade plasma-derived C1-INH (Berinert) was from CSL Behring (Danderyd, Sweden). Recombinant human PAD2 and PAD4 and biotin-phenylglyoxal (PG) were from Cayman Chemicals (Ann Arbor, MI, USA) (#10785, #10500, and #17450). For optimization experiments, in-house expressed PAD4 was used.

### 2.3 *In vitro* citrullination

C1-INH citrullinated according to the following protocol was used for all complement and contact system-related experiments. Both PAD2 and PAD4 are stored in 50 mM of HEPES, pH 8.0, with 200/300 mM of NaCl, 10% glycerol, and 1 mM of DTT. In order to be active, PAD enzymes require a reducing agent such as DTT and calcium. Citrullination was induced by incubating C1-INH with PAD2 or PAD4 in 50 mM of HEPES, pH 8.0, with 200 mM of NaCl, 10% glycerol, and 10 mM of CaCl_2_ (citrullination buffer) for 2 h at 37°C. PAD2 and PAD4 were 1:20 diluted in this reaction, and the final DTT concentration was consequently 50 μM. As negative controls, only PAD2 or PAD4 was incubated in citrullination buffer, or C1-INH was incubated in citrullination buffer containing 50 μM of DTT. In all assays, native C1-INH diluted in buffer without DTT and with DTT was compared in order to exclude any effect of the reducing agent. The presence of DTT at chosen concentration did not affect C1-INH (data not shown), and thus, this sample was used for comparison in all assays.

The following protocol was used to citrullinate C1-INH used in the ACPA ELISA. C1-INH was incubated with PAD2 and PAD4 in 100 mM of Tris-HCl (pH 7.5), 5 mM of DTT, and 10 mM of CaCl_2_ using 100 U of enzyme per mg C1-INH. The reaction was incubated for 2.5 h at 37°C. As negative controls, C1-INH, PAD2, and PAD4 were incubated separately in the same buffer.

### 2.4 Identification of citrullinated residues in C1-INH by LC-MS/MS

Samples were denatured and reduced in 8 M of urea and 50 mM of Tris-HCl, containing 10 mM of DTT, pH 8. After 60 min, the samples were alkylated by the addition of 30 mM of iodoacetamide. The samples were incubated in the dark for 60 min before the reaction was quenched by adding 10 mM of DTT. Finally, the samples were diluted 10 times with 100 mM of ammonium bicarbonate. Samples were treated with either endoproteinase Glu-C sequencing grade (Roche, Basel, Switzerland) or proteomics grade trypsin (Sigma-Aldrich, St. Louis, MO, USA) for 16 h at 37°C. The resulting peptides were micro-purified using Empore™ SPE C18 Disks packed in 10-µl pipette tips.

Liquid chromatography–tandem mass spectrometry (LC-MS/MS) was performed using an EASY-nLC 1200 system (Thermo Scientific) connected to an Orbitrap Eclipse Tribrid Mass Spectrometer (Thermo Scientific). Peptides were trapped on a 2-cm column (100-μm inner diameter) and separated on a 15-cm analytical column (75-μm inner diameter). Both columns were packed in-house with ReproSil-Pur C18-AQ 3 μm resin (Dr. Maisch GmbH, Ammerbuch-Entringen, Germany). Peptides were eluted using a flow rate of 250 nl/min and a 35-min gradient from 5% to 45% phase B (0.1% formic acid and 80% acetonitrile). The collected MS files were converted to mascot generic format (MGF) using Proteome Discoverer (Thermo Scientific). The data were searched against the human proteome (uniprot.org) and a common contamination database using a local Mascot Server (Matrix Science, Boston, MA, USA). The following settings were used: MS error tolerance of 10 ppm, MS/MS error tolerance of 0.02 Da, trypsin or Glu-C as protease, and carbamidomethyl as fixed modification. In addition, the following variable modifications were allowed: citrullination (R), deamidation (NQ), and oxidation (M). All spectra were manually inspected to confirm the presence of a citrullinated residue.

The relative amount of unmodified reactive site was determined based on the extracted ion chromatogram (XIC) of the Glu-C generated peptide AAAASAISVA**R**TLLVFE. The intensity of the reactive site containing peptide was normalized to the summed intensity of the two non-Arg containing peptides AVLGDALVDFSLKLYHAFSAMKKVE (918.1629 *m*/*z*, 688.8740 *m*/*z*, and 551.3007 *m*/*z*) and QALSPSVFKAIME (710.8789 *m*/*z*) within the same LC-MS/MS analysis. The normalized intensities were used to calculate the amount of unmodified reactive site in the PAD2/4-treated samples relative to non-treated C1-INH. All XIC data were obtained using Skyline 22.2 (28).

### 2.5 Detection of citrullinated C1-INH with PG-biotin by Western blotting and ELISA

Citrullinated proteins can be detected using PG-biotin (7). The PG-biotin probe reacts with citrulline at acidic pH, whereas at neutral pH, it reacts with Arg. MaxiSorp 96-well plates (Nunc) were coated with goat anti-human C1-INH (Complement Technologies, Tyler, TX, USA; #A240), diluted 1,000× in phosphate-buffered saline (PBS) overnight at 4°C. The plates were blocked with 3% fish-gelatin (Norlands Products, Jamesburg, NJ, USA; #HP-03) in 50 mM of Tris-HCl and 150 mM of NaCl, pH 8.0 (quench), for 3 h at 37°C. Citrullinated and native C1-INH (10 μM), PAD2 and PAD4 enzymes, and the negative control alpha1-antitrypsin (α1-AT, plasma purified in-house) were diluted in 50 mM of HEPES, pH 7.6, and 20% trichloroacetic acid (TCA); thereafter, 200 μM biotin-PG was added, and the mixtures were incubated for 30 min at 37°C. The reaction was quenched with 100 mM of citrulline, and the proteins were precipitated by 30-min incubation on ice. The samples were centrifuged at 14,000 *g* for 15 min at 4°C, and the pellets were washed twice in ice-cold acetone. The pellets were dried at 100°C for 5 min; resuspended in 20 mM of HEPES, pH 7.6, containing 100 mM of NaCl and 100 mM of Arg; and sonicated in a sonicator bath for 20 min. The MaxiSorp plate was washed with 50 mM of Tris-HCl and 150 mM of NaCl pH 8.0, and the samples, diluted in quench, were added to the plates and incubated at 37°C for 60 min. The citrullinated proteins, labeled with the biotin-PG probe, were detected using streptavidin–horseradish peroxidase (HRP) (R&D Systems, Minneapolis, MN, USA; #DY998) diluted in quench and incubated at 37°C for 60 min. The samples were developed with TMB ONE (Kem-En-Tech, Taastrup, Denmark; #4380) for 15 min, the reaction was stopped with 0.5 M of H_2_SO_4_, and the absorbances were measured at 450 and 620 nm in Cytation-5 multi-mode reader (BioTek, Winooski, VT, USA). For detecting citrullinated C1-INH by Western blotting, 2 μM of citrullinated C1-INH was used, and the proteins were treated in the same way as for the detection by ELISA, except that Laemmli sample buffer containing 25 μM of DTT was added, and samples were boiled for 5 min before sonication for 5 s. The samples were run on precast sodium dodecyl sulfate–polyacrylamide gel electrophoresis (SDS-PAGE) gels (Bio-Rad, Hercules, CA, USA) (14) and blotted using Trans-Blot Turbo (Bio-Rad). After blocking the membrane with quench, the citrullinated proteins were detected with streptavidin–HRP as for the ELISA and developed with Immobilon western chemiluminescent HRP substrate (Millipore, #WBKLS0500) using a CCD camera.

### 2.6 Binding of C1-INH to Alexa Fluor 647-labeled C1s

C1s (Complement Technologies, #A104) was labeled with Alexa Fluor 647 (AF647) using a microscale labeling kit (Thermo Scientific, #A30009). C1s-AF647 was mixed with citrullinated and native C1-INH, PAD2, and PAD4 enzymes or with citrullination buffer ± DTT in TBS with 5 mM of CaCl_2_ alone, and samples were incubated at 37°C for 60 min. Laemmli sample buffer, without a reducing agent, was added, and the samples were boiled for 3 min. The samples were separated using an Any kD SDS-PAGE gel (Bio-Rad) and analyzed on a CCD camera (Bio-Rad).

### 2.7 Dissociation of C1-complex

C1s (30 μg) was labeled with ^125^I (Perkin Elmer, Waltham, MA, USA; #NEZ033A) using iodination beads (Pierce, #28665). Breakable MaxiSorp plates (Nunc, #473768) were coated with 10 μg/ml of heat-aggregated IgG in 15 mM of Na_2_CO_3_ and 35 mM of NaHCO_3_, pH 9.6, and incubated overnight at 4°C. Between each step, the plate was washed with 50 mM of Tris-HCl and 150 mM of NaCl, pH 8.0, supplemented with 2 mM of CaCl_2_. The plate was blocked with 1% bovine serum albumin (BSA) in PBS at 37°C for 90 min. During blocking, the C1-complex was formed by mixing 2 μg/ml of C1s, 8 μg/ml of C1r (Complement Technologies, #A102), 20 μg/ml of C1q (Complement Technologies, #A099), and a trace amount of ^125^I-C1s in 2.5 mM of veronal-buffered saline containing 0.1% gelatin, 1 mM of MgCl_2_, 0.15 mM of CaCl_2_ and 2.5% dextrose (DGVB^++^) and incubated at 37°C for 60 min. The C1-complex was added to the plate and incubated at 37°C for 60 min. For dissociation of the C1-complex, various versions of 25 μg/ml of C1-INH and control proteins (α1-AT, prothrombin, or BSA) were diluted in DGVB^++^, added to the plated, and incubated at room temperature for 60 min. After the final wash, radioactivity was measured in individual wells in an Automatic Gamma Counter (Perkin Elmer).

### 2.8 Chromogenic assay for C1-INH activity

The Berichrom C1-inhibitor chromogenic assay kit was purchased from Siemens Healthineers, Solna, Sweden. Untreated C1-INH, PAD2 or PAD4-citrullinated C1-INH, only PAD2 or PAD4 (0.5, 1, 2, and 4 μg in a volume of 4 μl), or only citrullination buffer containing 50 μM of DTT were added to a 96-well plate containing 150 μl of C1s (reagent E) and incubated at 25°C for 10 min. The substrate (methoxycarbonyl-l-lysyl(ϵ-carbobenzoxy)-glycyl-l-arginyl-*p*-nitrolanilide (MeOC-Lys(ϵ-Cbo)-Gly-Arg-pNA) 5 mmol/L, 15 μl) was added to the wells and mixed, and the absorbance at 405 nm was measured every minute for 60 min in a Cytation-5 multi-mode reader.

### 2.9 Complement inhibition assays

Complement inhibition assays were performed as described previously (29) with minor adaptations. To test inhibition of the classical and lectin complement pathways, microtiter MaxiSorp plates were coated with 2.5 μg/ml of heat-aggregated IgG (Baxter Medical AB, Kista, Sweden) or 100 μg/ml of mannan from *Saccharomyces cerevisiae* (Sigma-Aldrich, #M7504) in PBS overnight at 4°C. After blocking with 1% BSA in PBS and washing with 50 mM of Tris-HCl and 150 mM of NaCl, pH 8.0, plates were incubated with normal human serum (NHS; 0.125% for classical pathway and 1% for lectin pathway) together with citrullinated and native C1-INH as well as PAD2 or PAD4 enzymes (NHS and the proteins were preincubated for 15 min on ice) diluted in 5 mM of veronal-buffered saline containing 0.1% gelatin (Sigma, #G1890), 1 mM of MgCl_2_, and 0.15 mM of CaCl_2_ (GVB^++^) and incubated at 37°C for 20 min. C4b deposition was detected with rabbit anti-human C4c antibody (Dako, Carpinteria, CA, USA; #Q0369) for 30 min at room temperature followed by swine anti-rabbit (Dako, #P0399) antibody conjugated with HRP for 60 min at room temperature, both diluted in blocking buffer. The plates were developed with TMB ONE for 10 min, the reaction was stopped with 0.5 M of H_2_SO_4_, and the absorbances were measured at 450 and 620 nm in a Cytation-5 multi-mode reader (29).

### 2.10 Chromogenic assay for contact system inhibition

Contact system proteins 320 nM of factor XII (FXII), 30 nM of factor XI (FXI), and 170 nM of plasma prekallikrein (PK) (Enzyme Research Laboratories, South Bend, IN, USA) were used in a chromogenic assay to determine if citrullinated C1-INH maintains its inhibitory effect. Based on the cleavage of chromogenic substrates specific to the contact system, inhibition by C1-INH would generate less of a signal in these assays. Two assays were set up in glass vials (DWK Life Sciences, Millville, NJ, USA) with one detecting activated FXII (FXIIa) and plasma kallikrein (KK) using chromogenic substrate PNAPEP-1902 (Cryopep, Montpellier, France), while the other detected activated FXI (FXIa) using chromogenic substrate S2366 (Chromogenix, Florham Park, NJ, USA). Citrullinated C1-INH samples and native C1-INH (Berinert) were serially diluted from 1 to 0.0625 µM in VBS++ (veronal-buffered saline containing 5 mM of Na-barbiturate, pH 7.4; 145 mM of NaCl; 0.15 mM of Ca^2+^; 0.5 mM of Mg^2+^) in glass tubes containing FXII/PK or FXII/PK/FXI prior to a 10-min incubation at 37°C. Thereafter, 100 µl of each sample was then transferred in duplicates onto a MaxiSorp 96-well plate (Nunc) and mixed with 50 µl of the corresponding chromogenic substrate for a 5-min incubation on a heated plate shaker at 37°C set to 250 rpm. Reactions were then stopped by the addition of 50 µl/well 20% citric acid before reading absorbance at 405 nm on a TECAN Spark plate reader (Männedorf, Switzerland).

### 2.11 Detection of autoantibodies to native and citrullinated C1-INH

Native and citrullinated C1-INH were diluted in coating buffer (30 mM of sodium carbonate and 70 mM of sodium bicarbonate) to 10 μg/ml and coated overnight at 4°C in MaxiSorp plates. To compensate for the content of PAD enzymes in the citrullinated samples, native C1-INH was mixed with either PAD2 or PAD4, each incubated separately in citrullination buffer (see above) directly before coating. Wells were washed with 50 mM of Tris-HCl, 150 mM of NaCl, and 0.1% Tween 20, pH 7.5 (Immunowash) and blocked with PBS containing 0.05% Tween-20 and 2% BSA (PBST/BSA). Plasma samples were 1:50 diluted in PBST/BSA, added to the wells, and incubated for 2 h at room temperature. After washing, wells were incubated for 1 h with 1:2,000 diluted HRP-conjugated polyclonal rabbit anti-human IgG (Dako, #P0214), washed again, and developed with TMB ONE. The reaction was stopped with 0.5 M of H_2_SO_4_, and the absorbances were measured at 450 and 620 nm in Cytation-5 multi-mode reader.

### 2.12 Statistical analyses

Data are presented as mean ± SD and analyzed with two-tailed one-way or two-way analysis of variance followed by Dunnett’s multiple comparison post-test. Statistical significance for non-parametric continuous data was calculated using the Mann–Whitney U test for two groups. Data are presented as medians with interquartile ranges. Correlations of non-parametric data were analyzed using Spearman’s rank-order correlation test. A statistical significance level of (*p*-value) <0.05 was defined as statistically significant, and *p*-values are displayed as *p < 0.05, **p < 0.01, ***p < 0.001, and ****p < 0.0001. Data were analyzed and graphed using GraphPad Prism v9.5.1 for Mac OS X (GraphPad Software) and JMP Pro 17 (SAS Institute) software.

## 3 Results

### 3.1 C1-INH was citrullinated using PAD2 and PAD4

**Figure 1:**
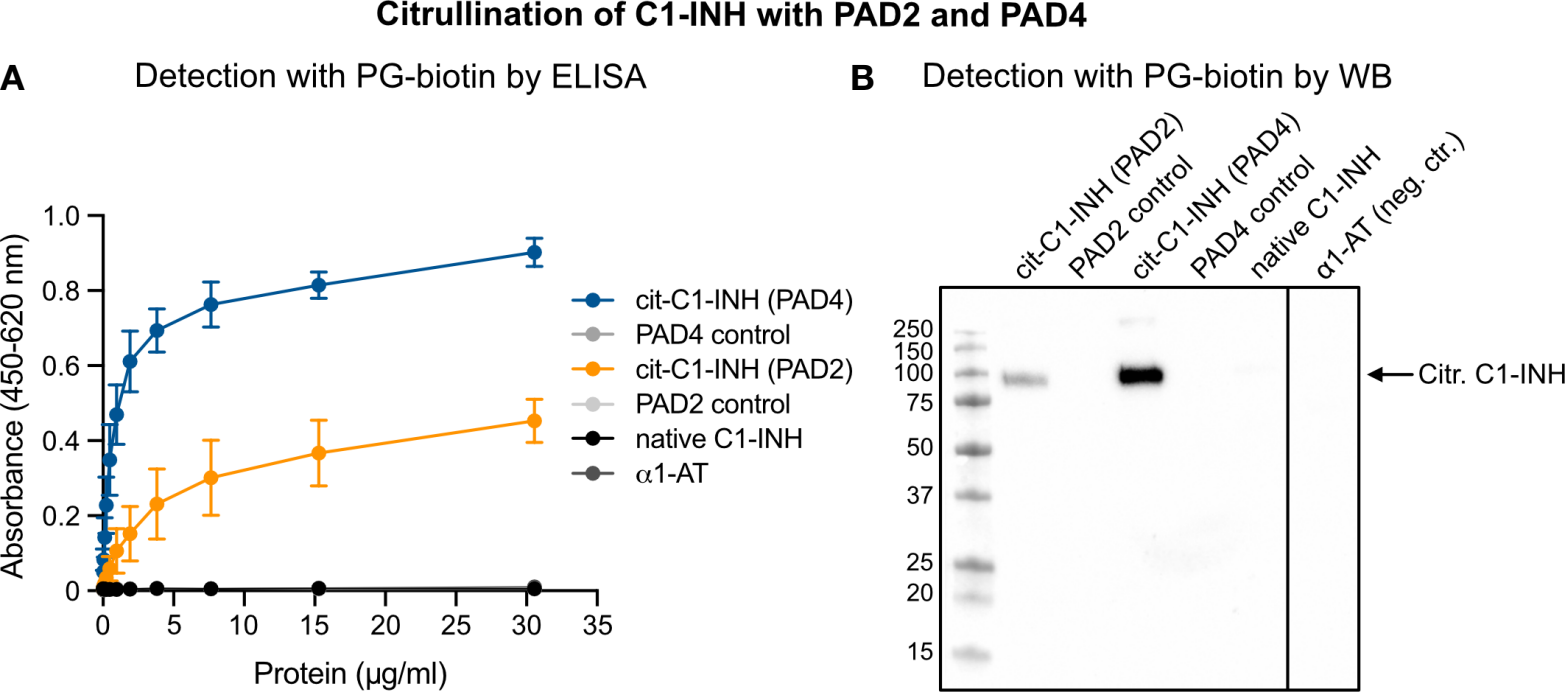
Confirmation of C1-INH citrullination by PAD4 and PAD2 using the PG-biotin probe on ELISA and Western blotting. C1-INH was incubated with PAD2 or PAD4 under reducing conditions and in the presence of 8 mM of CaCl_2_. Citrullination of C1-INH was confirmed using the biotin-PG probe at low pH and visualized in ELISA **(A)** and Western blotting **(B)**.

The detection of citrullinated proteins is challenging since the mass change is less than 1 Da. However, phenylglyoxal-based probes react specifically with a citrulline residue under acidic conditions by covalently labeling citrullinated proteins with fluorophore or biotin (PG-biotin). In the latter case, biotinylation can be used as a surrogate for antibodies in immunoassays. Recombinant C1-INH was incubated with PAD2 and PAD4 under reducing conditions and in the presence of 10 mM of calcium. *In vitro* citrullination of the C1-INH was detected using the PG-biotin probe and confirmed by ELISA (**Figure 1A**) and Western blotting (**Figure 1B**). Neither native C1-INH nor the PAD enzyme nor the negative control α1-AT reacted with the PG-biotin probe.

### 3.2 C1-INH was citrullinated at seven distinct Arg residues

Matured C1-INH contains 12 Arg residues, which are potential substrate sites for PAD2 and PAD4. However, these sites cannot be distinguished by the PG-biotin assay. Thus, LC-MS/MS was employed to map the citrullination of the C1-INH. To ensure high sequence coverage, both trypsin and Glu-C endopeptidase were utilized for peptide generation. Through this approach, a total of seven distinct sites were identified as being modified by both PAD2 (**Table 2**) and PAD4 (**Table 3**) including the reactive site Arg residue of C1-INH. The native peptides were also identified for each citrullinated site. Based on the ion intensity (XIC) of the peptide containing the reactive site (E.AAAASAISVA**R**TLLVFE.V), the amount of unmodified peptide was estimated at 1.2% and 2.7% for PAD2 and PAD4, respectively. In this way, our LC-MS/MS analysis provided conclusive evidence that both PAD2 and PAD4 very efficiently citrullinated the Arg residue at the reactive site of C1-INH, rendering the inhibitor inactive against its physiologically important target proteases.

**Table 2:** PAD2-citrullinated positions in C1-INH.

Peptide identified	Mascot score*	Start position	End position	Modified position	Protease
K.IS**R**LLDSLPSDTR.L	79	274	286	276	Trypsin
K.T**R**MEPFHFK.N	41	308	316	309	Trypsin
K.H**R**LEDMEQALSPSVFK.A	82	365	380	366	Trypsin
K.FQPTLLTLP**R**IK.V	51	391	402	400	Trypsin
K.FPVFMG**R**VYDPR.A	63	488	499	494	Trypsin
K.FPVFMG**R**VYDP**R**.A	47	488	500	494, 499	Trypsin
E.MSKFQPTLLTLP**R**IKVTTSQDMLSIME.K	44	388	414	400	Glu-C
E.AAAASAISVA**R**TLLVFE.V	94	456	472	**466**	Glu-C

The modified Arg residues and the position of the reactive site are highlighted in bold.

*Individual ions score above 32 indicate identity or extensive similarity (p < 0.01).

**Table 3:** PAD4-citrullinated positions in C1-INH.

Peptide identified	Mascot score*	Start position	End position	Modified position	Protease
K.IS**R**LLDSLPSDTR.L	69	274	286	276	Trypsin
K.T**R**MEPFHFK.N	43	308	316	309	Trypsin
K.H**R**LEDMEQALSPSVFK.A	106	365	380	366	Trypsin
K.FQPTLLTLP**R**IK.V	32	391	402	400	Trypsin
K.FPVFMG**R**VYDPR.A	68	488	499	494	Trypsin
K.FPVFMG**R**VYDP**R**.A	50	488	500	494, 499	Trypsin
E.AAAASAISVA**R**TLLVFE.V	118	456	472	**466**	Glu-C

The modified Arg residues and the position of the reactive site are highlighted in bold.

*Individual ions score above 32 indicate identity or extensive similarity (p < 0.01).

### 3.3 Binding to C1s is impaired when C1-INH is citrullinated

**Figure 2:**
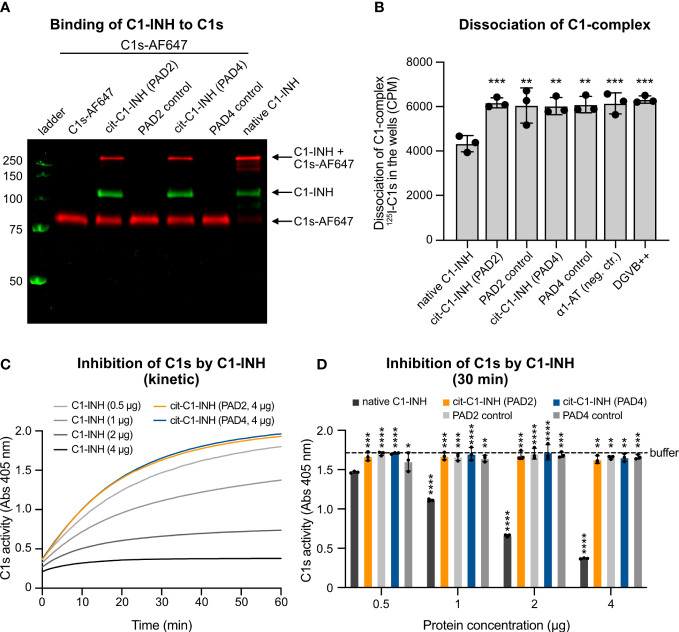
Citrullination of C1-INH impairs its interaction with C1s and prevents dissociation of the C1-complex. **(A)** C1s was labeled with AF647 and incubated with native and citrullinated C1-INH as well as only PAD2/PAD4 enzymes in TBS supplemented with 5 mM of CaCl_2_ at 37°C for 60 min. The samples are visualized on a non-reduced SDS-PAGE. **(B)** C1-INH citrullinated by PAD2 or PAD4 cannot dissociate the C1-complex from the IgG-coated microtiter plate. Native C1-INH can dissociate the C1-complex. Three individual citrullinations were analyzed in triplicate, and one-way ANOVA with Dunnett’s post-test was applied to determine statistically significant differences compared to C1-INH as control. Mean ± SD is shown. **(C, D)** Native C1-INH, PAD2/4-citrullinated C1-INH (0.5, 1, 2, or 4 μg per reaction each), or only citrullination buffer was mixed with C1s, and then a chromogenic substrate was added. The absorbance was measured every minute for an hour. **(C)** The activity of C1s over an hour. **(D)** The individual proteins at the 30-min time point. The buffer control (citrullination buffer) is shown as a dotted line. Two-way ANOVA with Dunnett’s multiple comparisons tests using 0.5 μg of C1-INH as control was applied to determine statistically significant differences between bars. Ns, not significant. Mean ± SD of three individual citrullinations are shown. *p < 0.05, **p < 0.01, ***p < 0.001, ****p < 0.0001. SDS-PAGE, sodium dodecyl sulfate–polyacrylamide gel electrophoresis.

When C1-INH reacts with C1s, a proteolytically inactive covalent inhibitory complex is formed. To visualize complex formation upon SDS-PAGE separation, C1s was labeled with AF647 and incubated with native and citrullinated C1-INH. Upon binding of native C1-INH, the intensity of free C1s-AF647 decreased, and the intensity of C1-INH/C1s-AF647 complexes increased (**Figure 2A**). However, when C1-INH was citrullinated by PAD2 or PAD4, the binding between C1-INH and C1s-AF647 was reduced, and the intensity of free C1s-AF647 was similar to the sample without the addition of C1-INH. PAD2 and PAD4 enzymes did not bind to C1s-AF647.

### 3.4 Citrullinated C1-INH cannot dissociate the C1-complex

The C1-complex consists of one C1q, two C1s, and two C1r molecules. The serpin C1-INH covalently binds to activated C1s and C1r, which effectively disassembles the C1-complex, releasing inactive C1-INH-C1r-C1s-C1-INH tetramer complexes, thereby inhibiting complement activation. A mixture of C1q, C1r, C1s, and trace amounts of ^125^I-radiolabeled C1s was added to IgG-coated MaxiSorp plates. Citrullinated and native C1-INH as well as control proteins were added, and the dissociation was measured by the remaining radioactivity in the wells. The native C1-INH was functionally active and could dissociate the C1-complex, whereas the citrullinated C1-INH was apparently inactive, as it could not disassemble the complex (**Figure 2B**). The PAD enzymes, the negative control α1-AT, and the buffer exerted no effect on the complex stability.

### 3.5 Citrullination of C1-INH impairs its activity toward C1s

The binding of citrullinated C1-INH to C1s was markedly reduced. To test whether citrullinated C1-INH can still inhibit C1s, we recorded C1s activity on a chromogenic substrate in the absence (buffer only) and the presence of native and citrullinated C1-INH. While native C1-INH reduced the C1s activity in a dose-dependent manner, the PAD-treated inhibitor exerted no effect on the rate of substrate hydrolysis, even at the highest amount (4 μg) used (**Figure 2C**). Of note, in this assay, PAD enzymes did not affect the kinetic of C1s inhibition by C1-INH, clearly indicating that prior citrullination of the Arg residues in the inhibitor active loop is a prerequisite to abrogate its ability to react with the protease. This hypothesis was fully corroborated by graphing the residual C1s activity at the 30-min time point (**Figure 2D**).

### 3.6 Citrullination of C1-INH impairs its inhibition of the classical and lectin complement pathways

**Figure 3:**
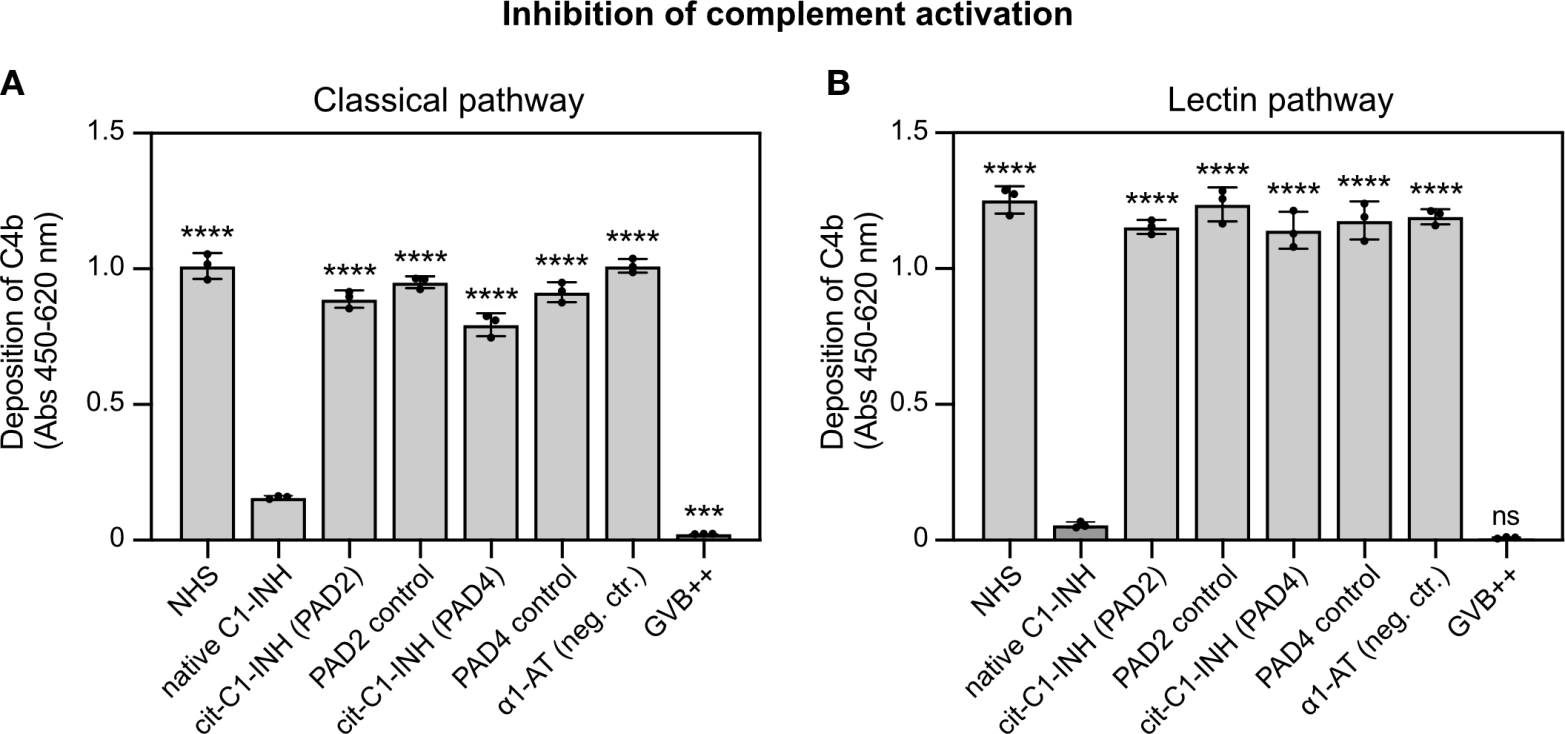
C1-INH inhibition of the classical and lectin complement pathways is impaired upon citrullination. **(A)** Heat-aggregated IgG (2.5 μg/ml) or **(B)** mannan (100 μg/ml) was coated on MaxiSorp 96-wells plates. Pooled normal human serum (0.125% in panel A; 1.0% in panel B) was pre-incubated with untreated and citrullinated C1-INH, or α1-AT as control (0.15 mg/ml) or only GVB^++^ for 15 min on ice, followed by complement deposition assays and ELISA detection. Three individual citrullinations were analyzed in duplicate, and one-way ANOVA with Dunnett’s post-test was applied to determine statistically significant differences compared to C1-INH as control. Mean ± SD is shown. ***p < 0.001; ****p < 0.0001; ns, not significant.

To investigate whether the citrullinated C1-INH is able to inhibit complement activation, MaxiSorp plates were coated with aggregated human IgG (classical pathway) and mannan (lectin pathway). Native and citrullinated C1-INH and controls proteins were added together with normal human serum, and C4b deposition was quantified. For the classical (**Figure 3A**) and lectin pathways (**Figure 3B**), native C1-INH was able to inhibit C4b deposition, whereas both PAD2 and PAD4 treatment of C1-INH significantly impaired the inhibition of C4b deposition. The incubation with only PAD2, PAD4, or the negative control α1-AT had no effect.

### 3.7 Citrullination of C1-INH impairs its ability to inhibit the contact system

**Figure 4:**
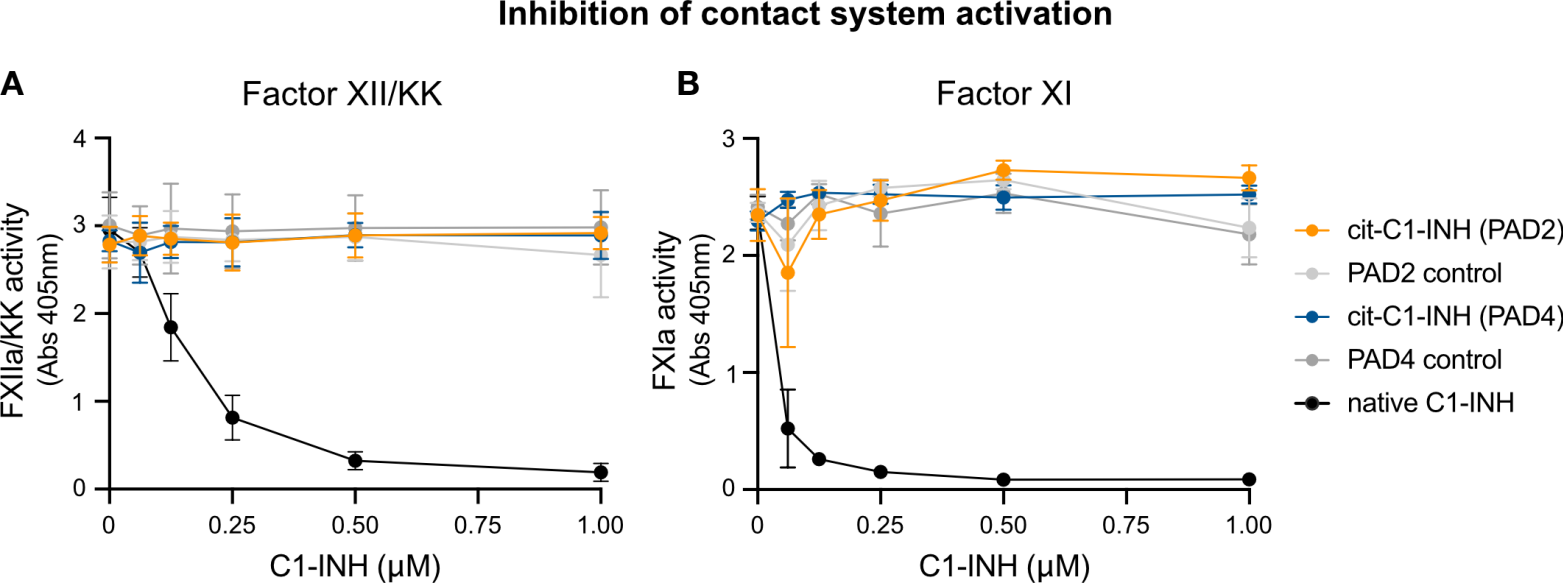
Chromogenic assay demonstrating the effect of C1-INH citrullination toward contact system activity. Native C1-INH, PAD2/4-citrullinated C1-INH, lone PAD2/4, and C1-INH in citrullination buffer + DTT were incubated with contact system components (FXII/PK or FXII/PK/FXI). These mixtures were incubated at 37°C in glass tubes for 10 min prior to a 5-min incubation with the respective FXIIa/KK **(A)** or FXIa **(B)** chromogenic substrate. Absorbance was measured at 405 nm.

To study if citrullinated C1-INH can inhibit components of the contact system, chromogenic assays specific for FXIIa/KK and FXIa were used. Upon contact with a glass surface, FXII is auto-activated to FXIIa, which in turn activates PK to KK and FXI to FXIa. Various concentrations of native and citrullinated C1-INH were added to FXII/PK and FXI in glass vials, and the activity of KK and FXIa was determined with corresponding chromogenic substrates. While native C1-INH reduced the activity of KK (**Figure 4A**) and FXa (**Figure 4B**) in a dose-dependent manner, PAD2 or PAD4 citrullinated C1-INH had no effect on the activity of the contact system components. Of note, PAD2 and PAD4 enzymes alone did not have any effect. This finding is in keeping with the lack of C1s inhibition by PAD-treated C1-INH, confirming our assertion that the citrullinated inhibitor is inactive.

### 3.8 Citrullination of C1-INH increases autoantibody reactivity

**Figure 5:**
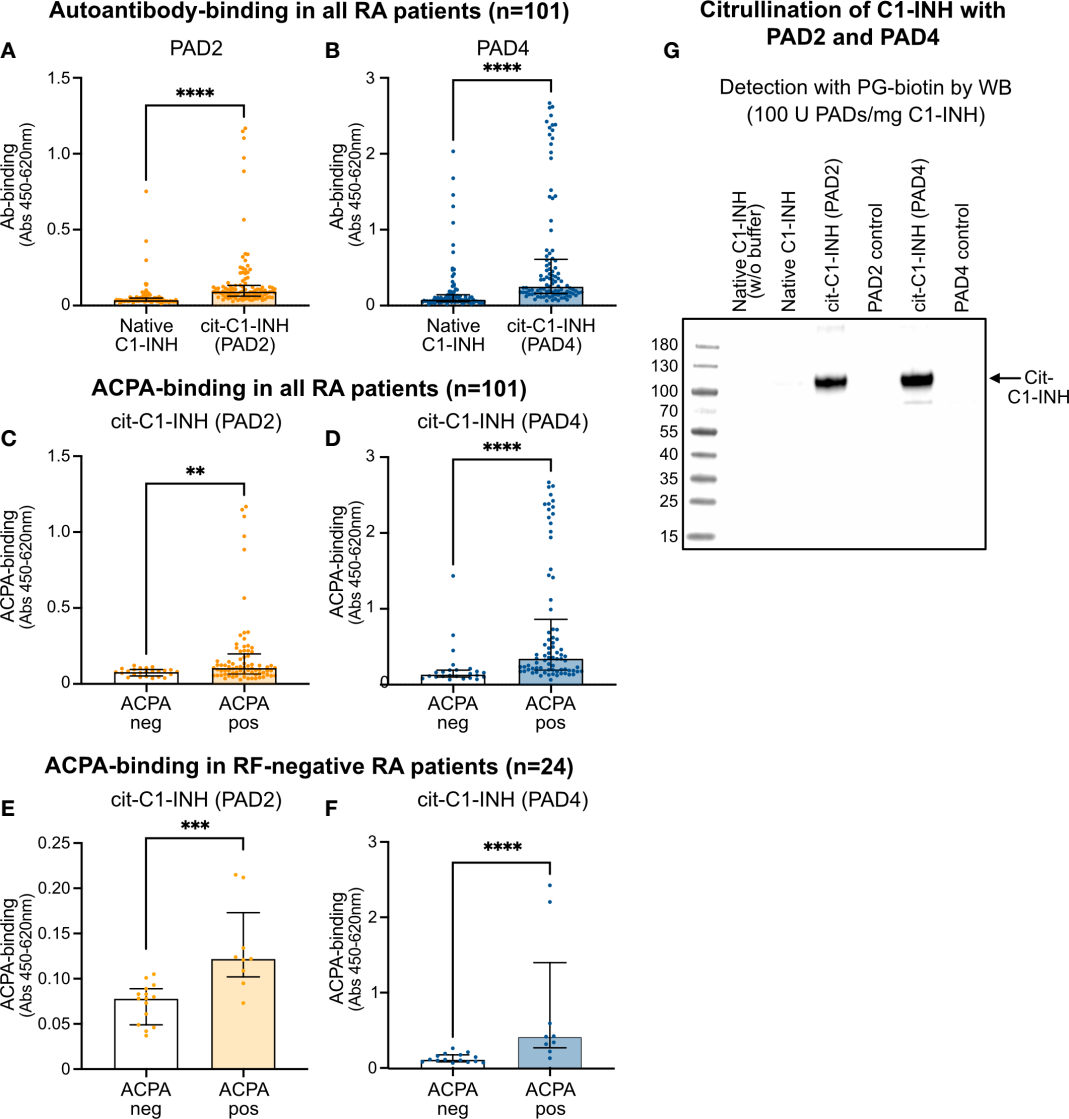
ELISA confirming ACPA reactivity to citrullinated C1-INH. **(A, B)** RA patient samples were added to microtiter plates coated with native and PAD2-citrullinated **(A)** as well as PAD4-citrullinated **(B)** C1-INH. Autoantibody binding was detected using HRP-conjugated rabbit anti-human IgG and compared between ACPA-negative and ACPA-positive samples for PAD2-citrullinated **(C, E)** and PAD4-citrullinated C1-INH **(D, F)** for all RA patient samples **(C, D)** as well as for merely RF-negative samples **(E, F)**. Data are presented as median plus interquartile range, and significance was calculated using Mann–Whitney U and Kruskal–Wallis rank-sum tests. Citrullination of C1-INH using 100 U of the PAD enzymes per 1 mg of protein was confirmed using the biotin-PG probe visualized by Western blotting **p < 0.01, ***p < 0.001, ****p < 0.0001 **(G)**. ACPA, anti-citrullinated protein antibody; RA, rheumatoid arthritis; HRP, horseradish peroxidase; RF, rheumatoid factor.

A direct ELISA was used to investigate the autoantibody reactivity in 101 RA patient samples against native, PAD2-citrullinated, and PAD4-citrullinated C1-INH. Most samples had very little reactivity against native C1-INH but significantly increased reactivity against PAD2-citrullinated C1-INH (**Figure 5A**) and even more increased reactivity against PAD4-citrullinated C1-INH (**Figure 5B**). The native C1-INH was mixed with the same amount of PAD2 (**Figure 5A**) and PAD4 (**Figure 5B**), which was present in the citrullination reaction. In this way, plates were coated with the same amount of native and citrullinated C1-INH. Notably, the native C1-INH and the PAD enzymes were each incubated separately in citrullination buffer and mixed before the addition to the plate.

Importantly, significantly more autoantibody reactivity was observed in ACPA-positive than in ACPA-negative patient samples for both PAD2-citrullinated (**Figure 5C**) and PAD4-citrullinated C1-INH (**Figure 5D**), while the reactivity against PAD4-citrullinated C1-INH seemed even stronger than against PAD2-citrullinated C1-INH. Significantly more autoantibody binding in RF-positive patients than in RF-negative patients was observed for PAD4-citrullinated C1-INH (*p* = 0.0022), but not for PAD2-citrullinated C1-INH (**Table 4**). Notably, analyzing merely the RF-negative patient samples, still more autoantibody reactivity was observed against the ACPA-positive than ACPA-negative samples for both PAD2-citrullinated (**Figure 5E**) and PAD4-citrullinated C1-INH (**Figure 5F**). By Western blotting, using the PG-biotin probe, it was confirmed that when using 100 U of the PAD enzymes per 1 mg protein, both PAD2 and PAD4 efficiently citrullinated the C1-INH (**Figure 5G**).

**Table 4:** Correlation of autoantibody reactivity against PAD2- and PAD4-citrullinated C1-INH with clinical features.

Clinical feature	ACPA reactivity against PAD2-citrullinated C1-INH		ACPA reactivity against PAD4-citrullinated C1-INH	
	Correlation coefficient r_s_	*p*-Value	Correlation coefficient r_s_	*p*-Value
Age	−0.1053	0.2946	0.1755	0.0792
Sex	–	0.1851	–	0.9931
Disease duration	−0.0789	0.4332	**0.3383**	**0.0005**
Smoking	–	0.1509	–	0.5094
ACPA status	–	**0.0036**	–	**<0.0001**
RF status	–	0.3621	–	**0.0022**
CRP at sampling	**0.2920**	**0.0034**	0.1648	0.1031
ESR at sampling	**0.2597**	**0.0094**	0.1670	0.0984
DAS28	0.1588	0.1165	**0.2743**	**0.006**
HAQ	0.0757	0.4543	0.1692	0.0924
Cortisone treatment	–	0.9918	–	0.4793

For correlations with continuous variables, Spearman’s rank-order correlation test was used, and Spearman’s correlation coefficient r_s_ and the p-values are listed. For associations with categorical variables, Mann–Whitney U and Kruskal–Wallis rank-sum tests were used, and the p-values are listed. Significant p-values (p < 0.05) and corresponding correlation coefficients are highlighted in bold. ACPA, anti-citrullinated protein antibody; RF, rheumatoid factor; CRP, C-reactive protein; ESR, erythrocyte sedimentation rate; HAQ, health assessment questionnaire.

The autoantibody reactivity to PAD2- and PAD4-citrullinated C1-INH did not associate with sex, smoking, or cortisone treatment and did not correlate to the health assessment questionnaire (**Table 4**). However, ACPA binding to PAD4-citrullinated C1-INH increased significantly with disease duration (*p* = 0.0005) and correlated significantly with the disease activity score DAS28 (*p* = 0.006, **Table 4**). ACPA reactivity against PAD2-citrullinated C1-INH correlated significantly with CRP levels (*p* = 0.0034) and ESR (*p* = 0.0094, **Table 4**). The same trend was observed for autoantibody reactivity to PAD4 but did not reach significance.

## 4 Discussion

In the current study, we show that citrullination of the C1-INH with PAD2 and PAD4 inactivates its function and thus abrogates its ability to inhibit the classical and lectin complement pathways as well as the plasma kallikrein–kinin system. This is apparently due to the conversion of the Arg residue present in the inhibitory active site, which is exposed on the reactive center loop of C1-INH, to citrulline, as all tested proteases are strictly specific for the Arg residue in the P1 position of the hydrolyzed peptide bond (7). However, the citrullination of additional Arg residues on the surface of the C1-INH molecule may also affect its function and antigenicity but has most likely a rather minor predictable effect on the inhibitory activity.

RA is a chronic inflammatory autoimmune disease mainly affecting the joints characterized by swelling, pain, and stiffness, which might progress to bone and joint destruction and impaired physical function (30). Immune cell infiltration into the synovium and the presence of autoantibodies are hallmarks of RA. Immune complexes of autoantibodies and their corresponding joint antigens activate the classical complement pathway leading to the generation of anaphylatoxins and membrane attack complex (MAC) that in turn attract more immune cells and cause the release of pro-inflammatory cytokines and proteases, leading to tissue damage (31). It is likely that the kallikrein–kinin system is also involved in the pathogenesis of RA since its activated components are found in the synovial fluid and various symptoms are related to the molecular reactions of the system (24, 32). C1-INH is among the most abundant protease inhibitors present in systemic blood circulation. It has multi-functional roles as the key inhibitor of the complement and contact systems (23).

Dysregulated citrullination is the essential driver for the production and maintenance of ACPA, which are present in more than 75% of RA patients and used as a diagnostic marker (4). Furthermore, while PAD dysregulation is observed in numerous inflammatory diseases including RA, ACPA is generally restricted to RA, and high titers correlate with disease severity (33). Thus, it is important to understand the source and mode of action of the citrullinating enzymes in RA. Under physiologic conditions, nanomolar intracellular calcium concentrations, the oxidizing environment, and probably protein cofactors are limiting the function of PAD enzymes. Low amounts of citrullinated proteins are efficiently cleared, and the load of immunogenic proteins is insufficient to drive an ACPA immune response. However, under pathologic conditions including dramatically increased calcium levels and a reducing environment, PADs are overactivated. Active PAD enzymes are found in RA synovial fluid, suggesting synovial cells and/or infiltrated immune cells as PAD sources. Deamidation of Arg residues can occur intracellularly but also extracellularly, and over 100 citrullinated proteins, among them C1-INH and other serpins, were so far identified in RA synovial fluid of which approximately one-third are extracellular proteins (7). Thus, PAD enzymes must be released into the synovial fluid. Neutrophils are reported to be the most abundant immune cell in the synovial fluid (34), and they are suggested as the major source of both intracellular and extracellular citrullination, the latter by releasing PADs (35, 36).

Various forms of cell death are suggested to play a role in the generation of citrullinated autoantigens in RA. Leukotoxic hypercitrullination occurs upon membranolytic damage of neutrophils induced by either the host or bacterial pore-forming proteins (37). Host pore-forming cytolytic proteins such as complement-derived MAC and perforin, released by natural killer cells and cytotoxic T cells, induce calcium influx and osmotic lysis. Even sublethal amounts of these pore-formers can increase the intracellular calcium concentration sufficiently to hyper-activate PADs (38–40). An initial ACPA response triggers the immune system further to generate more MAC and perforin, and a large number of dying neutrophils likely maintains a constant release of active PADs, which sustain the hypercitrullination in the RA joint (41). Pro-inflammatory cytokines and anaphylatoxins are additionally released, which recruit even more immune cells to the joints, and the vicious cycle results in chronic inflammation (42). NETosis, which is observed in various autoimmune disorders, was also thought to play a significant role in the generation and release of citrullinated proteins. However, recent studies revealed that citrullination is not a prerequisite for NET formation and that several proteins are already citrullinated in unstimulated NETs and only released, but not generated, upon NETosis (38).

Interestingly, only a few citrullinated proteins are targets for ACPA. The reason for this phenomenon is not yet completely understood, but it is known that the function of well-defined ACPA autoantigens such as α-enolase, vimentin, and fibrinogen is inactivated by *in vitro* hypercitrullination (43, 44), and it is hypothesized that certain citrullination sites are stronger drivers of immunogenicity. While fibrinogen is more extensively citrullinated by PAD2 than by PAD4 (45), the distinct forms of fibrinogen arising by citrullination with PAD4 are preferentially recognized by ACPAs (46). We observed stronger autoantibody reactivity to citrullinated than to native C1-INH, suggesting that the citrullinated protein is more immunogenic than the native form. Autoantibodies from anti-CCP-positive patient samples exhibited stronger reactivity against citrullinated C1-INH than anti-CCP-negative samples, clearly indicating that ACPAs constitute a major proportion of the reacting autoantibodies. RFs are autoantibodies against the Fc region of IgG; they occur in various inflammatory conditions and are thus not specific to RA. However, they are found in 70%–80% of RA patients and are highly cross-reactive, which can cause false-positive results in sandwich immunoassays (47). In our cohort, 72% of the RA patient samples were RF-positive. Since we still detected stronger reactivity to citrullinated C1-INH in ACPA-positive than in ACPA-negative samples when merely analyzing RF-negative patient samples, we postulate that there is a true-positive binding of ACPAs to citrullinated C1-INH. This is strengthened by the observation that RF-positive samples did not bind significantly stronger to PAD2-citrullinated C1-INH than RF-negative samples. For PAD4-citrullinated C1-INH, there was a significant difference, but it was much less pronounced than for the ACPA status.

ACPAs reacted with increased efficiency toward PAD4-citrullinated C1-INH than to PAD2-citrullinated C1-INH even though C1-INH was equally well citrullinated by both enzymes. This suggests that PAD4-citrullinated C1-INH is likewise PAD4-citrullinated fibrinogen more immunogenic than their PAD2-citrullinated counterparts, which might be due to different citrullination patterns by PAD2 and PAD4 of the additional Arg residues present in C1-INH. However, its ability to inhibit the complement and kallikrein–kinin systems was similarly abolished regardless of which PAD was used to modify the inhibitor. This is clearly due to the exposed nature of the Arg residue in the active site of the serpin, thus making it an easy target for citrullination. Apart from the active site, six additional Arg residues were found to be citrullinated in both PAD2- and PAD4-treated C1-INH. The applied mass spectrometric analysis allowed a quantitative comparison of the citrullination efficiency of the active site, which was almost completely modified by both PAD enzymes, which might explain why both PAD2- and PAD4-citrullinated C1-INH lost their inhibitory effect on the complement and contact systems to the same degree. Even though six additional Arg residues were found citrullinated in both PAD2- and PAD4-treated C1-INH, it is conceivable that the PAD4 enzyme modified these sites more efficiently than PAD2, contributing to the observed difference in their immunoreactivity with ACPA.

ACPA reactivity toward PAD4-citrullinated C1-INH increased with disease duration and correlates to the disease activity measured by the DAS28 score. ACPA binding to PAD2-citrullinated C1-INH seems to correlate more to the current inflammatory state. These findings are in line with the above-described activation of the complement pathways due to the inhibition of C1-INH and the release of pro-inflammatory cytokines and other acute phase reactants (such as CRP and ESR), which is clinically detected by the increased number of swollen and tender joints and higher DAS28 score. The presence of ACPA can maintain this process for years, which can explain the strong correlation with the disease duration but not age. Noticeably, the ACPA-positive/ACPA-negative status does usually not change during disease progression, which suggests that RA in the presence or absence of ACPA might be two distinct subtypes of the disease (48).

In addition, *in vitro* studies have shown that the ACPAs from RA patients activate both the classical and lectin pathways of complement, thereby further exacerbating both the inflammatory and autoimmune response (49). Citrullination of the C1-INH thus seems to render the protein more arthritogenic by two mechanisms, its inhibitory function is abolished, and it might become a target for ACPAs, which both contribute to the pathogenesis of RA.

We showed that citrullinated C1-INH was not able to bind the serine protease C1s and inhibit its activity. It is most probable that citrullinated C1-INH cannot bind C1r, MASP-1, and MASP-2 for which it is also the major natural inhibitor (21, 22). Citrullination of the C1-INH also abrogated its ability to dissociate the C1-complex and consequently inhibit C4b deposition *via* the classical and lectin pathways. It has been recently shown that citrullination of the P1-arginine, present in the reactive center loop of many serpins, generally inhibits their function (7). Serpins react with serine proteases through a reactive center loop. Serpins bind their targets irreversibly since they undergo a conformational change upon cleavage, which traps them in an inactive acyl-enzyme complex. We have confirmed that citrullination of the C1-INH indeed abolishes its activity, which may contribute to the uncontrolled complement activation observed in RA.

It is likely that other complement proteins and especially the serine protease C1r, C1s, MASP-1, and MASP-2 that exhibit an exposed P1 Arg in their activation loop might also be citrullinated in RA. Earlier proteomic analyses revealed the citrullinated form of various complement proteins such as C3, C4, C5, C6, C7, C9, C1r, and the complement inhibitor factor H in the serum and/or synovium of RA patients (7, 50). However, nothing is yet known about the impact of citrullination on the function of these proteins, and one can just speculate that it might further contribute to the pathogenesis of RA and that it is an interesting topic for future investigations.

The inhibitory effect of C1-INH on the contact system components FXIIa, plasma KK, and FXIa was also strongly reduced by citrullination. Activation of kallikrein leads to the formation of bradykinin, which causes major hallmarks of inflammation, edema, redness, pain, and heat. Hereditary or acquired deficiency of C1-INH both results in angioedema symptoms. Hereditary angioedema (HAE) arising from mutations in the SERPING1 gene encoding C1-INH is a rare autosomal dominant disease that can lead to life-threatening edemas (51), and the treatment with recombinant C1-INH is approved for acute and prophylactic management. Interestingly, bradykinin has been measured in RA synovial fluid and is increased compared to non-RA synovial fluid, and the expression of its receptors is altered in RA synoviocytes and neutrophils. In addition, kallikrein has been shown to activate one of the collagenases in RA patient synovial fluid (52), and collagen is one of the common autoantigens and targets of destruction in RA (53) and is used in RA models as a stimulating antigen that can induce the disease. Neutrophils express kallikrein and kininogens on their surface, and activation within the synovial fluid leads to the generation of kinins (54). The released kinins increase vasodilation and pain and induce the release of additional inflammatory mediators such as IL-1β and TNF-α, thus enhancing and perpetuating inflammation. Since we could show that citrullination of the C1-INH also abrogated its inhibitory effect on the contact system, it is very likely that kinin-mediated effects contribute to the inflammatory condition present in RA joints.

Understanding the underlying mechanisms of deregulated inflammation is vital in improving diagnostics and developing better therapeutic options. By increasing our knowledge of the typical RA citrullinome and the effects of citrullination on the complement and contact systems, our ongoing efforts will potentially open new avenues for therapy.

## Data availability statement

The raw data supporting the conclusions of this article will be made available by the authors, without undue reservation.

## Ethics statement

The studies involving human participants were reviewed and approved by Ethics Committee, Lund University, Sweden (Dnr 2013/846, Dnr 2017/582, Dnr 2021-03923). The patients/participants provided their written informed consent to participate in this study.

## Author contributions

AB, MM, KE, and BN designed the study. SN and LV performed the complement-related experiments and analyzed the results. MM performed the autoantibody reactivity experiments, analyzed the results, and calculated statistics on RA patient data. DE and KF performed contact system-related experiments and analyzed the results. CS and JE performed the mass spectrometry experiments and interpreted the results. MK provided the RA patient cohort and interpreted the corresponding results. MM, LV, SN, and AB interpreted the data and wrote the manuscript. EB and JP provided the human PAD4 enzyme. All authors contributed to the article and approved the submitted version.
